# Pre-pandemic assessment: a decade of progress in electronic health record adoption among U.S. hospitals

**DOI:** 10.1093/haschl/qxad056

**Published:** 2023-10-21

**Authors:** John (Xuefeng) Jiang, Kangkang Qi, Ge Bai, Kevin Schulman

**Affiliations:** Eli Broad College of Business, Michigan State University, East Lansing, MI 48824, United States; Harbert College of Business, Auburn University,Auburn, AL 36949,United States; Johns Hopkins Carey Business School, Baltimore, MD 21202, United States; Johns Hopkins Bloomberg School of Public Health, Baltimore, MD 21205, United States; Clinical Excellence Research Center, Stanford University School of Medicine, Palo Alto, CA 94304, United States; Stanford Graduate School of Business, Stanford, CA 94305, United States

**Keywords:** EHR, telemedicine, pandemic, EHR vendors

## Abstract

As the COVID-19 pandemic loomed, the adoption of electronic health records (EHRs) in US hospitals became a pivotal concern. This study provides a pre-pandemic assessment, highlighting a decade of progress in EHR adoption from 2009 to 2019, with the last available survey conducted from January to June of 2020. It delves into the current EHR adoption rates, variations across different hospital categories, the influence of major vendors, and the challenges in implementing these systems. The study found that basic EHR adoption surged from 6.6% to 81.2%, while comprehensive systems increased from 3.6% to 63.2%. Despite this growth, the findings point to enduring disparities among hospitals, a concentrated market share by 6 vendors (90%), and significant concerns regarding maintenance costs. These insights provide an invaluable snapshot of the state of EHR adoption at the brink of the pandemic, serving as a benchmark to assess hospitals’ readiness to utilize digital infrastructure in health care. The conclusions underscore the necessity for strategic policy interventions to encourage a competitive landscape and guarantee equitable access, ultimately strengthening the health care system's responsiveness to global health crises such as COVID-19.

## Introduction

The implementation of health information technology, including electronic health records (EHRs), has been crucial for improving health care efficiency and effectiveness, especially during the COVID-19 pandemic.^1^ Despite these advancements, existing EHR systems have come under scrutiny for their inadequacies in patient tracking and information sharing.^2^ The National Academy of Medicine has further emphasized the complexity of the problem, highlighting significant challenges in equity, interoperability, and the capacity to translate raw data into actionable insights.^3^ Taking stock of the current state of hospitals’ EHR systems is a first step to answer President Biden’s question to his science advisor: “How can we enable the rapid sharing, with patient consent, of health information to build a smarter and more effective health care system?”^4^

The American Recovery and Reinvestment Act (ARRA) of 2009 garnered bipartisan support for establishing a national, interoperable health information system.^5^ However, adoption rates were initially low. As of September 2008, only 7.6% and 1.5% of US hospitals had implemented basic and comprehensive EHR systems, respectively^2^ (A recent study suggests that the EHR adoption rate in hospitals as of 2008 could have been significantly higher if a more flexible definition of EHR adoption had been applied^6^). To bolster adoption rates, several public policies like the Medicare Promoting Interoperability Program, the Electronic Clinical Quality Measure, and the Hospital Inpatient Quality Reporting Program, were introduced. By 2014, adoption rates had climbed to 41% for basic EHR systems and 34% for comprehensive systems. Still, significant disparities existed across different categories of hospitals, including variations based on size, teaching status, ownership, location, and critical access status.^7^

The present study seeks to answer the following key questions: What are the most up-to-date EHR adoption rates? Have gaps in adoption between different hospitals narrowed? How does EHR adoption impact the ability to offer telemedicine services? Does vendor selection, given the market's limited major players, influence EHR adoption? And what challenges do hospitals encounter in implementing and using EHR systems? By answering these questions, we can better evaluate the preparedness of US hospitals in addressing the challenges posed by the COVID-19 pandemic.

**Figure 1.**
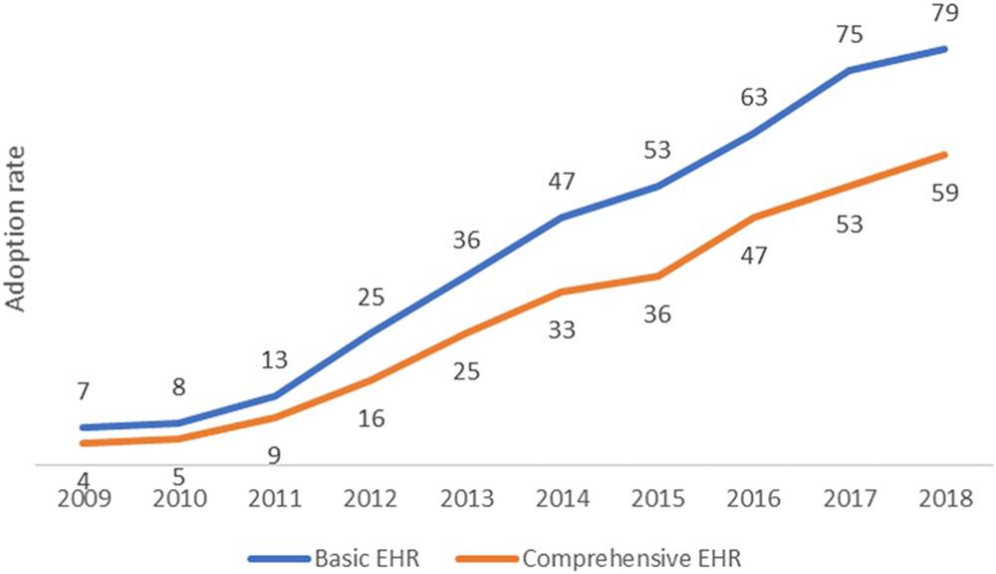
Adoption rate of basic and comprehensive EHRs in US hospitals, 2009–2019. Source: Authors’ analysis of data from the AHA annual survey and health care IT supplement survey. Abbreviations: AHA, American Hospital Association; EHR, electronic health record; IT, information technology.

Drawing on data from the American Hospital Association (AHA) that covers the years 2009 to 2019, with the survey for the 2019 data conducted from January to June of 2020, we discovered that nationwide adoption rates for basic EHR systems consistently climbed from 6.6% to 81.2%, while the rates for comprehensive EHR systems increased from 3.6% to 63.2% during the same period (Figure 1).

However, the disparities in EHR adoption rates among various types of hospitals have not diminished. Large hospitals exhibit a 7% higher comprehensive EHR adoption rate than medium-sized hospitals and are over 34% ahead of small hospitals. Meanwhile, rural hospitals trail urban areas in comprehensive EHR adoption by 12%. Furthermore, system-affiliated hospitals outpace independent ones, with differences of 15% in basic EHR and 22% in comprehensive EHR adoption rates. Similarly, teaching hospitals outperform non-teaching hospitals, leading by 14% in basic EHR adoption and 22% in comprehensive EHR adoption. These numbers reaffirm that the variations between different hospital categories have remained consistent, without significant narrowing since 2014.

At the onset of the COVID-19 pandemic (the 2019 survey was collected from January 2020 to June 2020), we observed that hospitals with EHR systems were more inclined to offer telemedicine services. This trend underscores the pivotal role of digital infrastructure in health care. Additionally, our findings reveal that 6 major vendors dominated 90% of the EHR market during the early part of 2020, a factor that strongly correlates with varying EHR adoption rates across hospitals. This market concentration and its implications point to potential areas of further examination.

Furthermore, we found that 65% of hospitals are planning to invest in enhancing the functionality of their EHR systems’ new releases, and more than half of the hospitals (53%) identify the persistent costs of maintaining and updating EHR systems as a considerable challenge, a concern that requires strategic consideration in future policy and management decisions.

The results of this study illuminate crucial insights into the state of EHR adoption across various hospital categories, shedding light on persistent disparities and unmet needs. The strong correlation between EHR adoption and telemedicine capabilities, along with the domination of the market by a few large vendors, underscores the importance of strategic policy interventions. These interventions should aim to foster a competitive landscape, ensure equitable access, and address the foundational challenges inherent in the current system architecture.

## Data and methods

### Data and sample

The AHA conducts an Annual Survey of Hospitals, profiling over 6200 hospitals and health care systems throughout the United States. In 2008, the AHA introduced a healthcare information technology (IT) supplement survey to assess the scope and degree of technology integration within hospitals.^8^ The timing of the 2019 survey, conducted from January 2020 to June 2020, has particular significance.^9^ As the COVID-19 pandemic unfolded, it led to a delay in the 2020 AHA IT survey, which was ultimately conducted in 2021 and released in 2022. This later survey focused on COVID-19–related information exchange and removed questions pertaining to EHR adoption status. Therefore, for our research on the trend of EHR adoption, the 2019 AHA IT survey represents the final available data. Conducted just as the full force of the pandemic was about to be felt, this timing uniquely aligns our study with an assessment of the readiness of health care systems’ digital infrastructure. Consequently, our analysis focused on the period from 2009 to 2019, encompassing 38 576 hospital-year observations, with an average of 3507 hospitals each year.

The AHA IT survey examines 23 EHR functionalities across 4 categories: clinical documentation, test and imaging results, computerized provider-order entry, and decision support. Following prior literature, we report both the individual EHR adoption rate and the average adoption rate of functionalities grouped under the “Basic EHR” and “Comprehensive EHR” systems.^2^ (We opted against weighting survey responses to maintain transparency and avoid strong assumptions. Regression of survey responses on hospital characteristics yielded a low adjusted *R*^2^ of 5%, indicating a weak relationship. Moreover, our unweighted comprehensive EHR adoption rate [33%] closely mirrored Adler-Milstein et al's^7^ weighted figure [34%]. Given this similarity and the added complexity of weighting, we chose a simpler, unweighted analysis.) We also linked hospitals’ EHR adoption to their ability to offer telemedicine, which is also available in the AHA IT survey. In addition, we gathered hospital characteristics such as ownership, teaching hospital designation, system affiliations, and location characteristics from the 2019 AHA annual survey.

### Statistical analysis

We first analyzed the adoption rate for individual functionalities as well as the average adoption rates for basic and comprehensive EHR systems from 2009 to 2019. We then examined the EHR adoption rate at the state level as of 2019, the last year when such data are available. Subsequently, we examined the basic and comprehensive EHR adoption rate across different hospital characteristics, such as ownership type, size, local status (rural or urban), system affiliation, and teaching hospital designation following the prior literature.^10-14^ Then, we reported the association between hospitals’ EHR readiness and their ability to provide telemedicine in 2019. EHR vendors have been found to exercise considerable influence on hospital technology strategy,^15^ so we also investigated the EHR adoption rates across different vendors. Then we described the planned changes that hospitals intend to make in the next 18 months and the primary obstacles faced in the execution and usage of an EHR system that meets the criteria of the Promoting Interoperability Programs—formerly known as the Meaningful Use Program. By conducting these analyses, we aim to identify the challenges that hospitals confront when seeking to further enhance patient engagement through electronic means.

## Results

### Adoption rates from 2009 to 2019

National adoption rates for all functionalities have steadily risen from 2009 to 2019, with the average adoption rate across functionalities reaching 91%, compared to 36% a decade prior (Table S1). ( The detailed adoption rate for each functionality and the categories of “Basic EHR” and “Comprehensive EHR” are presented. Basic EHR functionalities are a subset of comprehensive EHR functionalities, meaning that hospitals must first be basic EHR users and then adopt more advanced technologies, such as advanced directives (eg, do- not-resuscitate), diagnostic-test images (eg, electrocardiogram tracing), and all functionalities in the decision-support category, to become comprehensive EHR adopters. Notably, certain modules have experienced significantly faster growth in adoption rates, such as physicians’ notes, medications, and consultation requests, which began with adoption rates below 20% in 2009 and reached nearly 90% or above a decade later.

### EHR adoption by hospital characteristics

**Figure 2.**
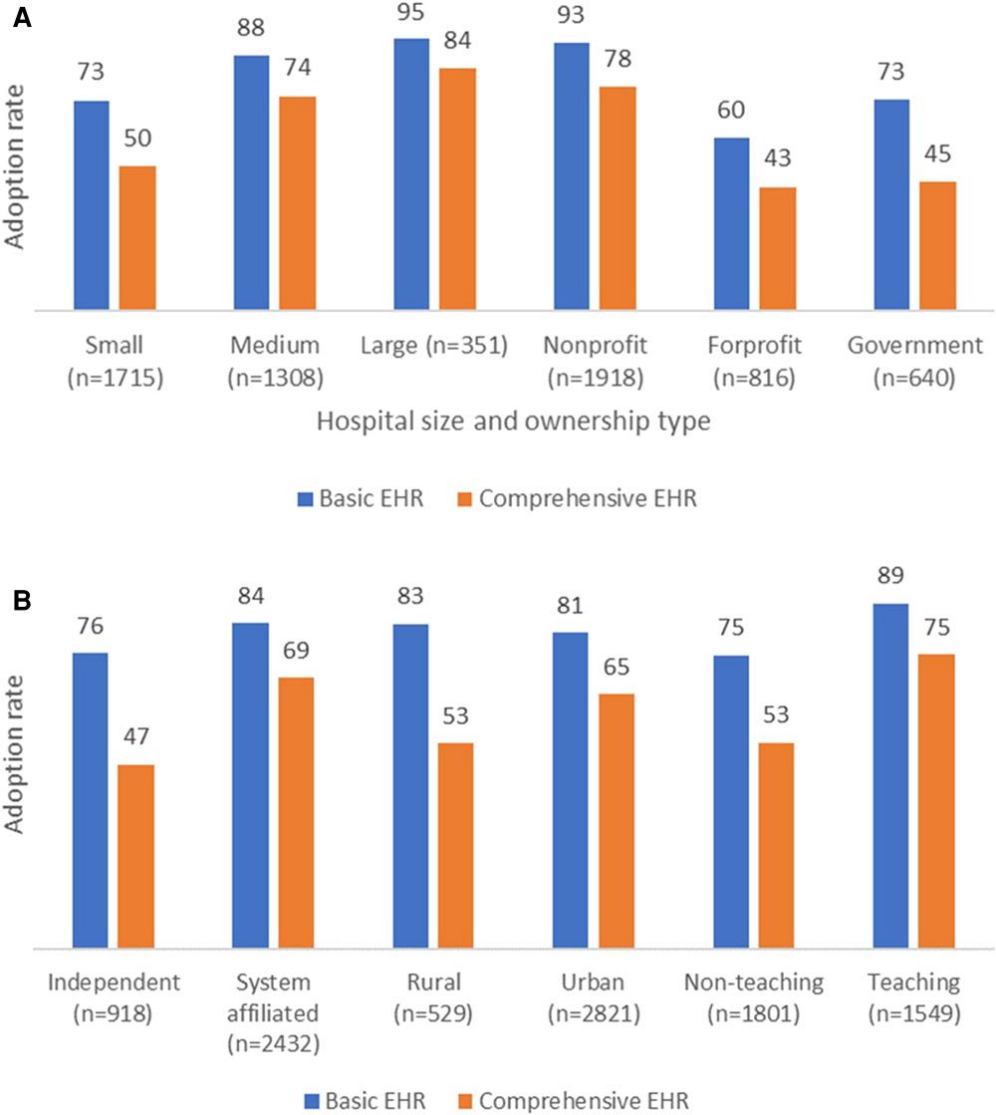
A, B: Adoption rate of basic and comprehensive EHRs in US hospitals, by different characteristics, as of January 2020 to June 2020. Source: Authors’ analysis of data from the 2019 AHA annual survey and health care IT supplement survey. Small hospitals (≤ 99 beds); medium hospitals (≥100 to ≤ 399 beds); large hospitals (≥400 beds). Abbreviations: AHA, American Hospital Association; EHR, electronic health record; IT, information technology.

For-profit hospitals have the lowest EHR adoption rates (60% for basic EHR and 43% for comprehensive EHR) compared with government hospitals (73% for basic EHR and 45% for comprehensive EHR) and nonprofit hospitals (93% for basic EHR and 78% for comprehensive EHR) (Figure 2). Larger hospitals have higher adoption rates than smaller hospitals. For instance, in 2019, 95% of large hospitals (≥400 beds) adopted basic EHR and 84% adopted comprehensive EHR, while medium-sized hospitals (100–399 beds) had adoption rates of 88% and 74%, respectively. In comparison, only 73% of small hospitals (≤99 beds) adopted basic EHR, and 50% adopted comprehensive EHR systems in 2019.

Rural hospitals have basic EHR adoption rates similar to urban areas (83% vs 81%, not statistically different) but lag in comprehensive EHR adoption rates (53% vs 65%) (Figure 2). System-affiliated hospitals have higher basic adoption rates (84% vs 76%) and comprehensive EHR adoption rates (69% vs 47%) than independent hospitals, potentially due to resource advantages or economies of scale. Teaching hospitals are more likely than non-teaching hospitals to adopt basic EHR (89% vs 75%) and comprehensive EHR (75% vs 53%). Compared with the statistics in 2014,^4^ the numbers indicate that the differences between various types of hospitals have not narrowed.

### EHR adoption and telemedicine offering

Figure S1 provides the percentage of hospitals offering telemedicine during the 2019 AHA IT survey period (January 2020 to June 2020). Hospitals with basic and comprehensive EHR systems are more likely to offer telemedicine services. Approximately 82% of hospitals with basic EHR offer telemedicine, while only 28% of hospitals without basic EHR do so. Additionally, approximately 87% of hospitals with comprehensive EHR offer telemedicine, compared to only 46% of hospitals without comprehensive EHR. Unreported correlation indicates a hospital's basic EHR adoption is highly correlated with its ability to offer telehealth service (correlation = 0.47).

### EHR vendors in early 2020

Out of the 3237 hospitals surveyed, a significant 90% use the services of the top 6 EHR vendors (Table S2).^12^ Consistent with prior research,^16^ Epic Systems Corporation is the leading player in the market, commanding a 35% market share, with Cerner following at a 23% market share.

The selection of vendors seems to correlate with variations in EHR adoption rates among different hospitals. For instance, hospitals using Epic's system demonstrate a high compliance rate, with 98% adhering to basic EHR adoption and 86% committing to comprehensive EHR adoption. Conversely, hospitals opting for Cerner's system present lower adoption rates—79% for basic and 61% for comprehensive EHR adoption.

These differences may stem from several underlying factors, such as the ease of implementation, integration capabilities with existing systems, user-friendliness, customer support, cost structures, and compliance with health care regulations. Additionally, the choice of vendor may have implications for how well hospitals are able to meet patient needs and regulatory requirements. A comprehensive examination of the characteristics of each system, along with qualitative feedback from users, might provide more in-depth insights into the reasons behind these disparities in adoption rates, and guide future decisions for health care providers in selecting EHR systems that best align with their specific needs and objectives.

### Intended changes and challenges in EHR implementation

**Figure 3.**
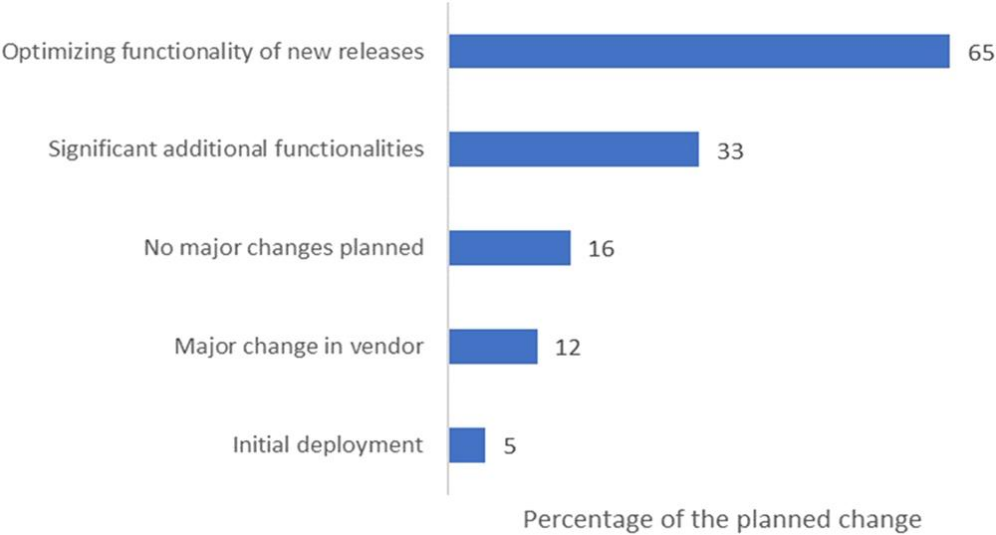
Percentage of hospitals that planned changes for primary inpatient EHRs in the next 18 months, as of January 2020 to June 2020. Source: Authors’ analysis of data from the AHA health care IT supplement survey. Abbreviations: AHA, American Hospital Association; EHR, electronic health record; IT, information technology.

Figure 3 depicts the changes that hospitals intend to make to their EHR systems over the next 18 months. A significant portion, 65% of the surveyed hospitals, plan to concentrate on enhancing the functionality of their new EHR system releases. Additionally, 33% of hospitals are aiming to add substantial new functionalities to their current EHR systems. Approximately 12% of hospitals are considering a significant shift in their EHR vendor, while 16% do not anticipate any substantial changes to their EHR systems.

**Figure 4.**
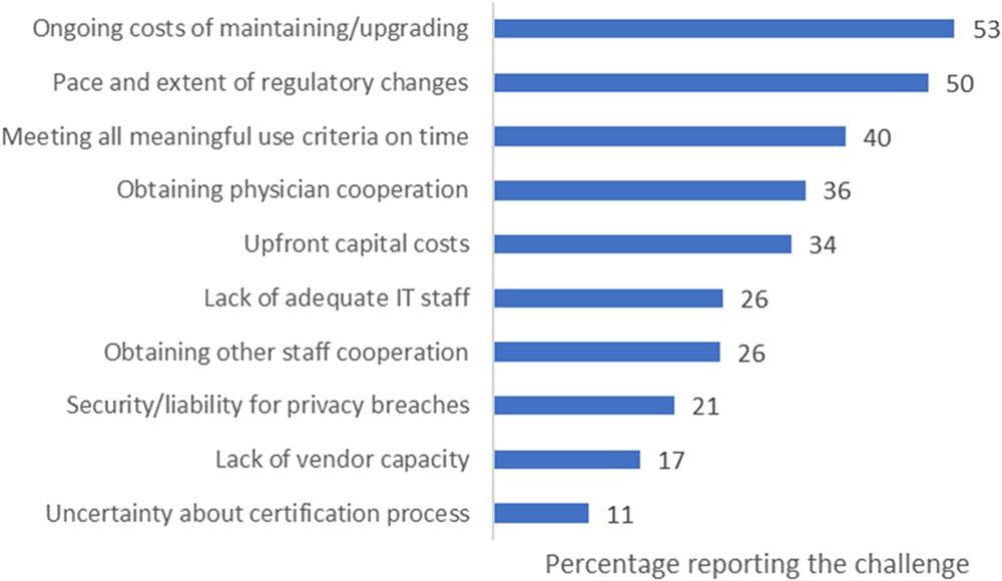
Primary challenges reported by hospitals in EHR implementation for promoting interoperability, as of January 2020 to June 2020. Source: Authors’ analysis of data from the AHA health care IT supplement survey. Abbreviations: AHA, American Hospital Association; EHR, electronic health record; IT, information technology.

Figure 4 details the main challenges hospitals encounter when implementing and using EHR systems that adhere to the requirements of the Promoting Interoperability Programs. Slightly more than half (53%) identified the persistent costs of maintaining and updating EHR systems as a significant obstacle. Just behind this, 50% of respondents faced issues in keeping pace with the fast and comprehensive shifts in other regulatory requirements.

In addition, 40% of the institutions found it difficult to meet all meaningful use criteria within the designated time frame, and 36% experienced issues with obtaining physician cooperation. A lack of upfront capital or financial resources was a concern for 34% of the respondents, while 26% had trouble securing cooperation from other staff members. The same percentage (26%) also reported a scarcity of sufficient IT staff. Furthermore, 21% were worried about security risks and potential liability arising from privacy breaches, and 17% highlighted a deficiency in vendor capacity.

## Discussion

The remarkable increase in EHR technology adoption among US hospitals over the past decade, as evidenced in our study, is a testament to the transformative impact of ARRA’s enactment in 2009. Nationwide adoption rates for basic EHR systems soared from a meager 6.6% to a robust 81.2%, while comprehensive EHR system adoption rates leaped from 3.6% to 63.2%. This significant growth highlights the success of federal efforts to digitize health care records.

Nevertheless, we must acknowledge the considerable disparities in EHR adoption rates among different types of hospitals. Larger, urban, nonprofit system hospitals have exhibited greater EHR adoption, likely benefiting from their financial capabilities to invest in electronic records system technology. Conversely, smaller, rural, and public (government) hospitals, often limited by financial constraints, are trailing in adoption rates. This divergence raises concerns about equitable access to advanced health care technology.

Our research provides critical insights into the intricate relationship between the adoption of EHR systems and vendor choices. Different vendors offer functionalities that can either promote or inhibit a hospital's capacity for digital engagement with patients. This issue gained acute significance at the onset of the COVID-19 pandemic; we discovered that many hospitals had inadequate EHR systems, limiting their capability for crucial data exchange with public health agencies and their efficiency in deploying telemedicine services. These findings should serve as a catalyst for future public policies aimed at leveling the EHR adoption landscape across various hospital types and enhancing EHR system maintenance and optimization. By strategically addressing these shortcomings, policymakers and health care leaders have a unique opportunity to forge a more resilient, adaptable health care system, better prepared for current and future public health crises.^17^

Our findings underscore that hospitals primarily grapple with the challenges of maintaining and updating their EHR systems. Simply allocating more resources to the existing, flawed architectures, as suggested by the Office of the National Coordinator (ONC) certification program, is unlikely to result in meaningful improvements. At its core, this is a business issue: the prevalent client-server architectures are hospital-centric rather than patient-centric, creating system bottlenecks. These architectures are particularly incompatible with emerging technologies and services such as telemedicine and artificial intelligence.^3^ The escalating maintenance costs further exacerbate the problem and pose a growing threat to health care systems. A pivot toward patient-centric, interoperable architectures is urgently needed. Such a shift would not only alleviate maintenance costs but also enable health care systems to effectively utilize emerging digital technologies. By making this transition, we can cultivate a health care ecosystem that is both resilient and agile, thereby better equipping it to tackle the complex challenges of today and the future.

## Supplementary Material

qxad056_Supplementary_Data
