# Supplementary material for: Pre-pandemic assessment: a decade of progress in electronic health record adoption among U.S. hospitals
Source: Health Aff Sch. 2023 Oct 21;1(5):qxad056. doi: 10.1093/haschl/qxad056 (PMC10986221; doi:10.1093/haschl/qxad056)
Supplement: qxad056_Supplementary_Data [file qxad056_Supplementary_Data.zip › Appendix Tables.docx]

**Table S1: Percentage of U.S. Hospitals Adopting Key EHR Functionalities, 2009 - 2019**

|  | **Basic** | **Comp.** | **2009** | **2010** | **2011** | **2012** | **2013** | **2014** | **2015** | **2016** | **2017** | **2018** | **2019** |
| --- | --- | --- | --- | --- | --- | --- | --- | --- | --- | --- | --- | --- | --- |
| **Clinical documentation (% of hospitals)** | | |  | | | | | | | | | | |
| Physician's notes | √ | √ | 16 | 18 | 24 | 35 | 47 | 58 | 67 | 77 | 83 | 92 | 94 |
| Nursing notes | √ | √ | 38 | 43 | 52 | 66 | 74 | 80 | 84 | 86 | 88 | 94 | 94 |
| Problem lists | √ | √ | 29 | 30 | 38 | 57 | 68 | 75 | 82 | 84 | 87 | 93 | 94 |
| Medication lists | √ | √ | 46 | 50 | 57 | 70 | 77 | 84 | 87 | 88 | 90 | 96 | 96 |
| Discharge summaries | √ | √ | 45 | 49 | 52 | 62 | 70 | 78 | 82 | 84 | 88 | 94 | 94 |
| Advanced directives |  | √ | 35 | 40 | 51 | 63 | 70 | 78 | 79 | 82 | 79 | 81 | 85 |
| **Test and imaging results (% of hospitals)** | | |  |  |  |  |  |  |  |  |  |  |  |
| Laboratory reports | √ | √ | 70 | 74 | 77 | 83 | 85 | 88 | 89 | 90 | 90 | 94 | 94 |
| Radiologic reports | √ | √ | 70 | 73 | 76 | 82 | 84 | 86 | 87 | 86 | 88 | 92 | 88 |
| Radiology images |  | √ | 66 | 71 | 74 | 80 | 81 | 83 | 83 | 83 | 88 | 89 | 87 |
| Diagnostic test results | √ | √ | 48 | 53 | 58 | 67 | 73 | 76 | 77 | 79 | 83 | 85 | 85 |
| Diagnostic test images |  | √ | 37 | 43 | 50 | 59 | 66 | 70 | 72 | 75 | 79 | 82 | 81 |
| Consultant reports |  | √ | 45 | 50 | 54 | 62 | 68 | 73 | 77 | 79 | 82 | 89 | 90 |
| **Computerized provider-order entry (% of hospitals)** | | | |  |  |  |  |  |  |  |  |  |  |
| Laboratory tests |  | √ | 22 | 23 | 32 | 48 | 62 | 76 | 81 | 84 | 87 | 93 | 93 |
| Radiology tests |  | √ | 21 | 22 | 31 | 48 | 62 | 75 | 79 | 83 | 86 | 92 | 92 |
| Medications | √ | √ | 18 | 21 | 30 | 48 | 62 | 76 | 82 | 84 | 87 | 94 | 94 |
| Consultation requests |  | √ | 16 | 18 | 27 | 43 | 56 | 71 | 76 | 80 | 82 | 89 | 89 |
| Nursing orders |  | √ | 21 | 23 | 32 | 49 | 63 | 76 | 82 | 83 | 87 | 92 | 93 |
| **Decision support** |  |  |  |  |  |  |  |  |  |  |  |  |  |
| Clinical guidelines |  | √ | 19 | 21 | 29 | 43 | 53 | 66 | 68 | 71 | 74 | 83 | 85 |
| Clinical reminders |  | √ | 23 | 26 | 33 | 46 | 56 | 67 | 70 | 72 | 77 | 86 | 87 |
| Drug-allergy alerts |  | √ | 45 | 49 | 57 | 69 | 78 | 83 | 86 | 87 | 90 | 95 | 96 |
| Drug–drug interaction alerts |  | √ | 44 | 48 | 56 | 68 | 77 | 83 | 85 | 86 | 89 | 94 | 95 |
| Drug–laboratory interaction alerts |  | √ | 32 | 37 | 44 | 56 | 65 | 71 | 74 | 77 | 81 | 86 | 88 |
| Drug-dose support |  | √ | 30 | 35 | 41 | 53 | 61 | 70 | 73 | 76 | 79 | 85 | 88 |
| **Average adoption rate** |  |  | 36 | 40 | 47 | 59 | 68 | 76 | 79 | 82 | 85 | 90 | 91 |
| **Basic EHR adoption (% of hospitals)** | | | 7 | 8 | 13 | 25 | 36 | 47 | 53 | 63 | 72 | 79 | 81 |
| **Comprehensive EHR adoption (% of hospitals)** | | | 4 | 5 | 9 | 16 | 25 | 33 | 36 | 47 | 53 | 59 | 63 |

**Source:** Authors’ analysis of data from AHA annual healthcare IT supplement survey.

**Notes:** The terms "Basic EHR adoption" and "Comprehensive (comp.) EHR adoption" refer to whether a hospital has replaced paper records in a certain number of functions, with Basic requiring replacement in nine specific functions, and Comprehensive requiring replacement in all 23 functions. The data used for this classification comes from the AHA IT supplement survey conducted annually between 2009 and 2019, resulting in 38,576 hospital-year observations. On average, each annual survey included responses from 3,507 hospitals.

**Table S2: EHR Vendor Market Share, 2019**

| **Vendor** | **Market Share** | **Basic EHR adoption** | **Comp. EHR adoption** |
| --- | --- | --- | --- |
| Epic Systems Corporation | 35% | 98% | 86% |
| Cerner Corporation | 23% | 79% | 61% |
| Meditech | 16% | 90% | 71% |
| Other | 10% | 42% | 18% |
| CPSI/Evident | 6% | 75% | 31% |
| Allscripts | 5% | 90% | 66% |
| MEDHOST | 4% | 63% | 50% |

**Source:** Authors’ analysis of data from AHA annual healthcare IT supplement survey.

**Notes:** The data is from the 2019 AHA IT supplement survey with 3237 hospitals in the survey. We report the top 6 individual EHR vendors’ market share, and the percentage of hospitals that use their equipment also adopt the basic and comprehensive (comp.) EHR. The percentage of Basic EHR adoption represents hospitals that adopted basic EHR and Comprehensive EHR adoption rate represent refer to whether a hospital has replaced paper records in a certain number of functions, with Basic requiring replacement in nine specific functions, and Comprehensive requiring replacement in all 23 functions.
