# Supplementary figures and images for: Pre-pandemic assessment: a decade of progress in electronic health record adoption among U.S. hospitals
Source: Health Aff Sch. 2023 Oct 21;1(5):qxad056. doi: 10.1093/haschl/qxad056 (PMC10986221; doi:10.1093/haschl/qxad056)

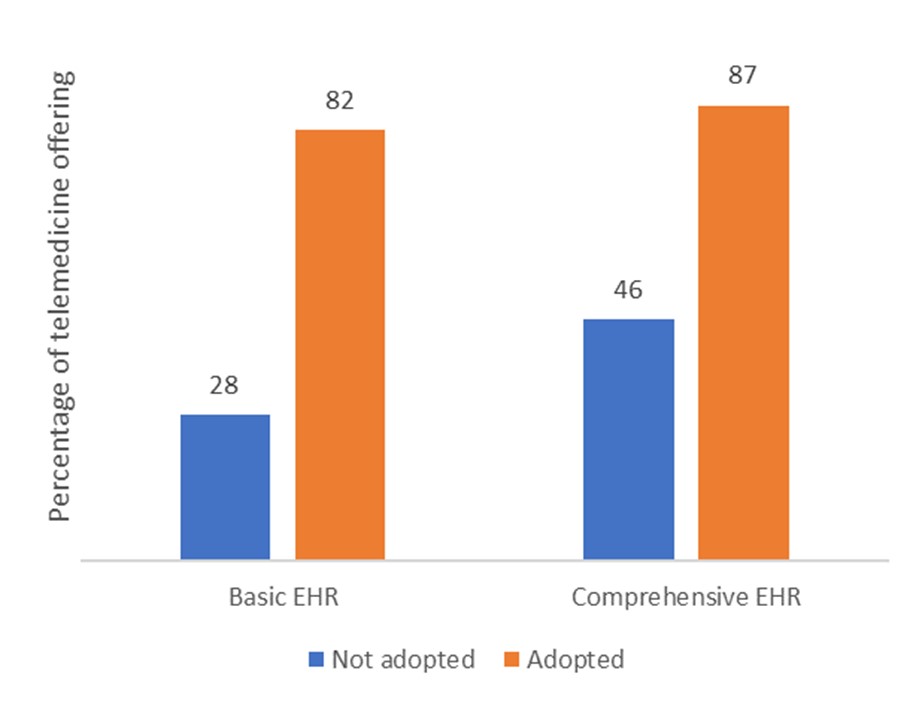

Supplement: qxad056_Supplementary_Data [file qxad056_Supplementary_Data.zip › Figure S1.jpg]
